# Brain atrophy pattern in de novo Parkinson’s disease with probable RBD associated with cognitive impairment

**DOI:** 10.1038/s41531-022-00326-7

**Published:** 2022-05-24

**Authors:** Javier Oltra, Carme Uribe, Barbara Segura, Anna Campabadal, Anna Inguanzo, Gemma C. Monté-Rubio, Jèssica Pardo, Maria J. Marti, Yaroslau Compta, Francesc Valldeoriola, Carme Junque, Alex Iranzo

**Affiliations:** 1 Medical Psychology Unit, Department of Medicine, Institute of Neurosciences, University of Barcelona, Barcelona, Catalonia Spain; 2 Institute of Biomedical Research August Pi i Sunyer (IDIBAPS), Barcelona, Catalonia Spain; 3 Research Imaging Centre, Campbell Family Mental Health Research Institute, Centre for Addiction and Mental Health (CAMH), University of Toronto, Toronto, Ontario Canada; 4 Centro de Investigación Biomédica en Red Enfermedades Neurodegenerativas (CIBERNED: CB06/05/0018-ISCIII), Barcelona, Catalonia Spain; 5 Parkinson’s Disease & Movement Disorders Unit, Neurology Service, Hospital Clínic de Barcelona, Institute of Neurosciences, University of Barcelona, Barcelona, Catalonia Spain; 6 Multidisciplinary Sleep Disorders Unit, Neurology Service, Hospital Clínic de Barcelona, Barcelona, Catalonia Spain

**Keywords:** Parkinson's disease, Magnetic resonance imaging

## Abstract

Rapid eye movement sleep behavior disorder (RBD) is associated with high likelihood of prodromal Parkinson’s disease (PD) and is common in de novo PD. It is associated with greater cognitive impairment and brain atrophy. However, the relation between structural brain characteristics and cognition remains poorly understood. We aimed to investigate subcortical and cortical atrophy in de novo PD with probable RBD (PD-pRBD) and to relate it with cognitive impairment. We analyzed volumetry, cortical thickness, and cognitive measures from 79 PD-pRBD patients, 126 PD without probable RBD patients (PD-non pRBD), and 69 controls from the Parkinson’s Progression Markers Initiative (PPMI). Regression models of cognition were tested using magnetic resonance imaging measures as predictors. We found lower left thalamus volume in PD-pRBD compared with PD-non pRBD. Compared with controls, PD-pRBD group showed atrophy in the bilateral putamen, left hippocampus, left amygdala, and thinning in the right superior temporal gyrus. Specific deep gray matter nuclei volumes were associated with impairment in global cognition, phonemic fluency, processing speed, and visuospatial function in PD-pRBD. In conclusion, cognitive impairment and gray matter atrophy are already present in de novo PD-pRBD. Thalamus, hippocampus, and putamen volumes were mainly associated with these cognitive deficits.

## Introduction

Rapid eye movement (REM) sleep behavior disorder (RBD) is a parasomnia characterized by vivid dreams, increased electromyographic activity during REM sleep associated with complex movements and loss of atonia^1^. RBD is a common symptom in Parkinson’s disease (PD) patients, with a prevalence of about 40%^2^. Also, isolated RBD (iRBD) is a prodromal symptom of PD and other synucleinopathies, with a rate of conversion to a clinically defined synucleinopathy up to 90% after 15 years of follow-up^3^. Results from postmortem studies in PD revealed pathological changes, with more diffuse and severe deposition of synuclein in patients with RBD symptoms^4^.

The diagnosis of RBD is performed by clinical history and video-polysomnography (vPSG) showing REM sleep with loss of atonia. When vPSG is not available the term probable RBD (pRBD) refers to individuals with clinical symptoms suggestive of RBD or fulfilling validated RBD validated questionnaires. A recent meta-analysis reported that in PD, the occurrence of RBD is associated with male sex, advance age, longer disease duration, increased Hoehn and Yahr (H&Y) stage, and with a higher Movement Disorder Society Unified Parkinson’s Disease Rating Scale (MDS-UPDRS) Part III score. The frequency of PD-pRBD increases with disease duration, H&Y stage, and MDS-UPDRS Part III score as well^5^. Furthermore, PD-pRBD has been associated with worse cognitive performance^6,7^ and more rapid cognitive decline^7^. Also, PD-RBD has been associated with a higher prevalence of mild cognitive impairment (MCI)^8^. Altogether, these results suggest that PD-RBD patients have different clinical and neuropsychological characteristic features and prognosis, being proposed as a specific PD subtype^9^.

Magnetic resonance imaging (MRI) studies may allow investigating the differences between PD with and without RBD. To the best of our knowledge, there are only three studies with de novo PD-pRBD patients in which quantified MRI was examined. They were performed with the Parkinson’s Progression Markers Initiative (PPMI) database, in which participants were classified according the RBD Screening Questionnaire (RBDSQ). One study performed with deformed-based morphometry (DBM), reported reduced volumes of pontomesencephalic tegmentum, medullary reticular formation, hypothalamus, thalamus, putamen, amygdala, and anterior cingulate in PD-pRBD compared with PD without probable RBD (PD-non pRBD) patients^10^. A second study showed that PD-pRBD had volume reduction in the putamen compared with PD-non pRBD patients by means of voxel-based morphometry (VBM)^11^. Lastly, the most recent study performed longitudinal analyses with a reduced sample and without control group. The results reported that in the baseline (de novo PD stage) the PD-pRBD group had thinning in the bilateral inferior temporal cortex compared with the PD-non pRBD group through cortical thickness analysis^12^.

Previous studies in advanced PD with small samples, comparing PD-RBD patients with PD-non RBD patients, have found volume decreases in the thalamus using volumetry and VBM^13^; decreased gray matter volume of the left posterior cingulate and hippocampus thought MRI volumetry^14^; and cortical thinning in the right perisylvian and inferior temporal cortices; as well as volume shape contraction in the putamen using cortical thickness and DBM approaches^15^.

In this context, the association between cognitive impairment and brain atrophy in de novo PD with RBD symptomatology has been poorly investigated. Furthermore, cortical thickness between-groups differences have never been explored in a large sample of de novo PD patients, including PD groups with and without pRBD and a healthy control group. The current work aims to examine subcortical and cortical measures of atrophy, concerning pRBD status, in a large sample of newly diagnosed drug naïve PD patients through MRI volumetry from global to deep gray matter (GM) nuclei segmentation, and cortical thickness analysis. Then, we aimed to find associations between structural abnormalities and cognitive impairments in PD-pRBD patients.

## Results

### Demographic and clinical characteristics

The final sample comprised of 79 PD-pRBD and 126 PD-non pRBD patients.

There were no differences between groups in sex, age, years of education, age of onset, disease duration, MDS-UPDRS Part III scores, and H&Y stage. PD-pRBD had higher RBDSQ, 15-item Geriatric Depression Scale (GDS-15) frequency of depression, Epworth Sleepiness Scale (ESS) total and frequency of sleepiness, Scales for Outcomes in Parkinson Disease (SCOPA-AUT) item 6 total and frequency of constipation, and MDS-UPDRS scores compared with PD-non pRBD; and higher RBDSQ, GDS-15 total, ESS total and frequency of sleepiness, SCOPA-AUT item 6 total and frequency of constipation, and lower University of Pennsylvania Smell Identification Test (UPSIT-40) scores compared with controls. Higher RBDSQ, higher SCOPA-AUT item 6 total and frequency of constipation, and lower UPSIT-40 scores were found in PD-non pRBD compared with controls (Table 1). Around 31% of the available MRI scans of PD patients were discarded after quality control (Supplementary Fig. 1).

**Table 1:** Demographic and clinical characteristics of PD-non pRBD, PD-pRBD, and HC.

	PD-non pRBD (*n* = 126)	PD-pRBD (*n* = 79)	HC (*n* = 69)	Test stats	*P*-value
Sex, male, *n* (%)	73 (57.9)	54 (68.4)	40 (58.0)	2.558^1^	0.278
Age, y, mean (SD)	62.2 (7.5)	64.3 (7.1)	62.6 (6.8)	2.126^2^	0.121
Education, y, mean (SD)	15.6 (2.9)	15.8 (3.0)	16.7 (2.6)	3.008^2^	0.051
Age of onset, y, mean (SD)	61.3 (7.4)	63.3 (7.1)	NA	1.908^3^	0.058
Disease duration, m, mean (SD)	10.5 (7.2)	11.0 (7.1)	NA	0.560^3^	0.576
RBDSQ, mean (SD)	2.6 (1.1)	6.8 (1.8)	1.7 (1.3)	300.998^4^	<0.001^5,6,7^
GDS-15, mean (SD)	2.0 (2.1)	2.7 (2.5)	1.5 (3.0)	4.335^2^	0.014^7^
Depressed, *n* (%)	13 (10.3)	16 (20.3)	7 (10.1)	4.924^1^	0.085^5^
ESS, mean (SD)	5.3 (3.1)	6.5 (3.6)	5.0 (3.2)	4.692^2^	0.010^5, 7^
Sleepy, *n* (%)	16 (12.8)	19 (24.1)	6 (8.8)	7.553^1^	0.023^5,6,7^
SCOPA-AUT item 6, mean (SD)	0.5 (0.6)	0.8 (0.7)	0.2 (0.4)	19.148^4^	<0.001^5,6,7^
Constipated, *n* (%)	60 (47.6)	51 (64.6)	10 (14.7)	37.832^1^	<0.001^5,6,7^
MDS-UPDRS, mean (SD)	29.4 (10.8)	35.7 (13.6)	NA	3.676^3^	<0.001
MDS-UPDRS Part III, mean (SD)	19.8 (8.0)	21.6 (9.1)	NA	1.478^4^	0.141
H&Y stage, *n*, 1/2/3	55/69/2	33/46/0	NA	1.398^1^	0.497
UPSIT, mean (SD)	22.2 (8.0)	19.6 (8.3)	34.1 (3.8)	84.620^4^	<0.001^6,7^

*RBDSQ* REM Sleep Behavior Disorder Screening Questionnaire, *GDS-15* 15-item Geriatric Depression Scale, *ESS* Epworth Sleepiness Scale, *MDS-UPDRS* Movement Disorder Society Unified Parkinson’s Disease Rating Scale, *H&Y* Hoehn & Yahr scale, *UPSIT* University of Pennsylvania Smell Identification Test, *PD-non pRBD* PD without probable RBD, *PD-pRBD* PD with probable RBD, *HC* healthy controls.

^1^The *Χ*^2^ test was used.

^2^Analysis of variance (ANOVA) followed by post hoc test corrected by Bonferroni was used.

^3^*t*-test was used.

^4^Analysis of variance (ANOVA) followed by post hoc test corrected by Games-Howell was used.

^5^Significant differences (*p* < 0.05) were found between PD-non pRBD and PD-pRBD.

^6^Significant differences (*p* < 0.05) were found between PD-non pRBD and HC.

^7^Significant differences (*p* < 0.05) were found between PD-pRBD and HC.

### Neuropsychological performance

**Fig. 1:**
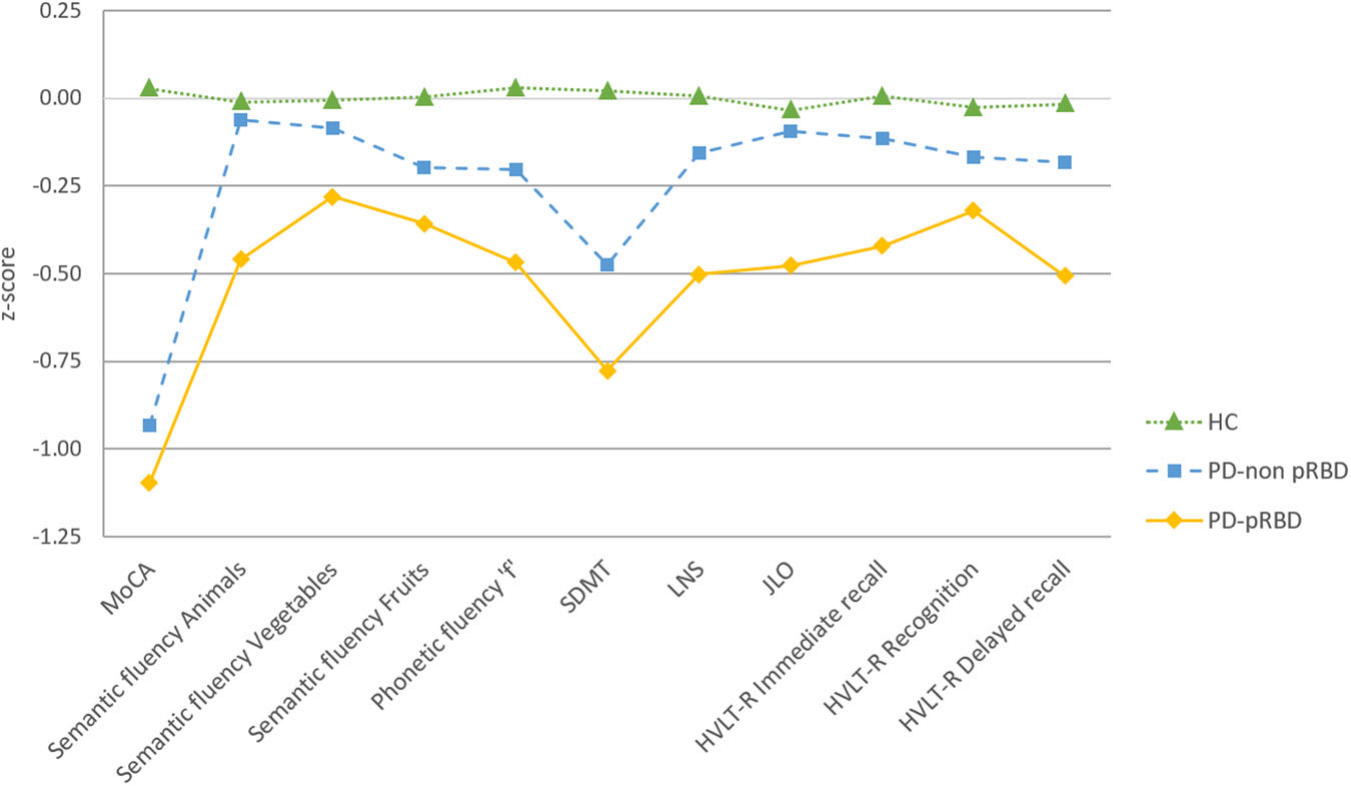
Neuropsychological performance. Tasks are indicated in the *x* axis. Group means in each task are presented as *z-*scores, as indicate in *y* axis. Data are presented as *z*-scores adjusted by age, sex and education. Lower *z*-scores indicate worse performance. Descriptive statistics of raw scores, as mean (SD), are available in Supplementary Table 1. Healthy controls in green, PD-non pRBD in blue, PD-pRBD in yellow. Healthy controls represented by filled triangles and a dotted line, PD-non pRBD by filled squares and a dashed line and PD-pRBD by filled rhombuses and a continuous line. MoCA Montreal Cognitive Assessment, SDMT Symbol Digit Modalities Test, LNS Letter-Number Sequencing, JLO Benton Judgment of Line Orientation, HVLT-R Hopkins Verbal Learning Test-Revised, HC healthy controls, PD-non pRBD PD without probable RBD, PD-pRBD PD with probable RBD.

PD-pRBD scored lower in semantic fluency and Benton Judgment of Line Orientation (JLO) than PD-non pRBD; when comparing them with controls, had lower Montreal Cognitive Assessment (MoCA), semantic fluency, phonetic fluency, Symbol Digit Modalities Test (SDMT), Letter-Number Sequencing (LNS), JLO, Hopkins Verbal Learning Test-Revised (HVLT-R) immediate and delayed recall scores. When comparing the PD-non pRBD group with the control group, MoCA and SDMT scores were lower in the former (Fig. 1, Supplementary Table 1). As supplementary, we performed analyses with the MDS-UPDRS score as a covariate, the PD-pRBD group had lower scores than the PD-non pRBD group in semantic fluency, LNS, and JLO (see Supplementary Table 2).

### Global and partial volume ratios

PD-pRBD had less left thalamus volume than PD-non pRBD. Atrophy of the left and right putamen, left hippocampus, and left amygdala was observed in PD-pRBD patients with respect to the control group. Finally, PD-non pRBD showed a decreased partial volume ratio in the right amygdala compared with the control group (Table 2). Additional analyses with the MDS-UPDRS score as a covariate were also performed. The PD-pRBD group showed decreased left thalamus and right pallidum volumes with respect to the PD-non pRBD group (see Supplementary Table 3).

**Table 2:** Global and partial volume ratios of PD-non pRBD, PD-pRBD, and HC.

	PD-non pRBD	PD-pRBD	HC	Test stats	*P*-value
Global atrophy volumes					
Cortical GM	28.6704 (2.16624)	28.7482 (2.51696)	29.6923 (2.09360)	5.020	0.007^1,2^
Subcortical GM	3.5597 (0.25621)	3.4926 (0.29242)	3.6037 (0.28252)	3.140	0.045^1^
Ventricular system	1.9743 (0.99272)	2.2429 (1.04248)	1.7940 (0.82571)	4.082	0.018^1^
Deep GM nuclei					
Left thalamus	0.4559 (0.04486)	0.4407 (0.04097)	0.4556 (0.04221)	3.464	0.033^3^
Right thalamus	0.4460 (0.04276)	0.4348 (0.04267)	0.4451 (0.04165)	1.841	0.161
Left caudate	0.2124 (0.02607)	0.2108 (0.02504)	0.2155 (0.02593)	0.636	0.530
Right caudate	0.2190 (0.02797)	0.2167 (0.02808)	0.2201 (0.02643)	0.311	0.733
Left putamen	0.2902 (0.03366)	0.2811 (0.03785)	0.2955 (0.03683)	3.124	0.046^1^
Right putamen	0.2883 (0.03211)	0.2816 (0.03791)	0.2955 (0.03659)	2.934	0.055^1^
Left pallidum	0.1280 (0.01427)	0.1259 (0.01687)	0.1267 (0.01498)	0.509	0.602
Right pallidum	0.1256 (0.01355)	0.1212 (0.01663)	0.1236 (0.01422)	2.159	0.117
Left hippocampus	0.2548 (0.02980)	0.2494 (0.02969)	0.2623 (0.02942)	3.507	0.031^1^
Right hippocampus	0.2619 (0.03127)	0.2606 (0.03106)	0.2699 (0.02957)	2.028	0.134
Left amygdala	0.1000 (0.01482)	0.0984 (0.01815)	0.1052 (0.01435)	3.710	0.026^1^
Right amygdala	0.1089 (0.01427)	0.1088 (0.01671)	0.1146 (0.01425)	3.813	0.023^2^
Left accumbens	0.0294 (0.00680)	0.0300 (0.00732)	0.0299 (0.00597)	0.233	0.792
Right accumbens	0.0317 (0.00662)	0.0317 (0.00700)	0.0322 (0.00583)	0.156	0.856
Brainstem	1.3904 (0.12004)	1.3618 (0.12659)	1.3870 (0.12034)	1.435	0.240

*GM* gray matter, *PD-non pRBD* PD without probable RBD, *PD-pRBD* PD with probable RBD, *HC* healthy controls.

Data are presented by groups as mean (SD). Analysis of variance (ANOVA) followed by post hoc test corrected by Bonferroni was used.

^1^Significant differences (*p* < 0.05) were found between PD-pRBD and HC.

^2^Significant differences (*p* < 0.05) were found between PD-non pRBD and HC.

^3^Significant differences (*p* < 0.05) were found between PD-non pRBD and PD-pRBD.

PD-pRBD compared with controls showed decreases in total cortical and subcortical GM volume ratios, as well as an increment in the ventricular system volume ratio. PD-non pRBD showed a decrease in total cortical GM volume ratio with respect to controls (Table 2).

Descriptive statistics of global and partial volumes in mm^3^ are shown in Supplementary Table 4.

### Regression models predicting cognition in PD-pRBD

Exploratory results of correlations are shown in Supplementary Tables 5 and 6. We performed two regression analyses in the PD-pRBD group, one using global measures of atrophy and the other using the volumes of deep GM nuclei (Table 3). Complementary, the regression analyses were also performed in the whole PD sample (Supplementary Tables 7 and 8).

**Table 3:** Multiple regression results of global and partial volume ratios as predictors of performance in neuropsychological tasks in the PD-pRBD group.

	Model 1 Global MRI measures		Model 2 Partial MRI measures	
	Variables	t-stat (*P*-value)	Variables	t-stat (*P*-value)
MoCA	Subcortical GM	3.277 (0.002)	Right putamen	2.124 (0.037)
			Left hippocampus	2.021 (0.047)
Semantic fluency Animals	No prediction model		No prediction model	
Semantic fluency Fruits	Non-significant model		Non-significant model	
Phonetic fluency ‘f’	Cortical GM	3.256 (0.002)	Left putamen	2.476 (0.015)
SDMT	Subcortical GM	2.465 (0.016)	Left hippocampus	2.765 (0.007)
LNS	Non-significant model		No prediction model	
JLO	Non-significant model		Left thalamus	2.261 (0.011)
HVLT-R immediate recall	No prediction model		Non-significant model	
HVLT-R delayed recall	No prediction model		Non-significant model	

*GM* gray matter, *MoCA* Montreal Cognitive Assessment, *SDMT* Symbol Digit Modalities Test, *LNS* Letter-Number Sequencing, *JLO* Benton Judgment of Line Orientation, *HVLT-R* Hopkins Verbal Learning Test-Revised.

When using global volume ratios to predict cognition in PD-pRBD, cortical GM explained a proportion of the phonetic fluency variance (*R*2 = 0.121; adjusted *R*2 = 0.110; *F* = 10.600; *p* = 0.002); subcortical GM significantly explained a proportion of the MoCA variance (*R*2 = 0.122; adjusted *R*2 = 0.111; *F* = 10.740; *p* = 0.002), and the SDMT variance (*R*2 = 0.0740; adjusted *R*2 = 0.062; *F* = 6.075; *p* = 0.016).

When using partial volume ratios to predict cognition in PD-pRBD, right putamen and left hippocampus explained a proportion of the MoCA variance (*R*2 = 0.180; adjusted *R*2 = 0.158; *F* = 8.317; *p* < 0.001); left putamen explained a proportion of the phonetic fluency variance (*R*2 = 0.074; adjusted *R*2 = 0.062; *F* = 6.133; *p* = 0.015); left hippocampus explained a proportion of the SDMT variance (*R*2 = 0.091; adjusted *R*2 = 0.079; *F* = 7.647; *p* = 0.007); and left thalamus explained a proportion of the JLO variance (*R*2 = 0.082; adjusted *R*2 = 0.070; *F* = 6.819; *p* = 0.011).

### Cortical thickness

PD-pRBD had cortical thinning in the right superior temporal gyrus compared with controls (MNI coordinates: *x*, *y*, *z* = 44, 17, −28; cluster size = 2461.25 mm^2^; *t-stat* = 2.836, *p* = 0.010) (Supplementary Fig. 2). None significant correlation was found between the cortical thickness of the significant cluster and cognition in the group PD-pRBD (see Supplementary Table 6 for significant results in the whole PD group).

## Discussion

In this study, we described the neuropsychological and brain structural characteristics in a large sample of PD-pRBD patients as well as the relation between cognitive impairment and measures of brain atrophy. PD-pRBD showed significant differences from controls and PD-non pRBD in neuropsychological tests and deep GM structures. Cognitive impairment of this subgroup of PD patients is related to diffuse global brain atrophy and reduced volumes of basal ganglia, thalamus, and hippocampus. Regarding clinical measures, remarkably, the PD-pRBD group had more presence of depression, sleepiness, constipation, and higher MDS-UPDRS total score than the PD-non pRBD group. Altogether, these findings suggest that the presence of RBD symptomatology is related to a more severe PD phenotype.

Patients with PD-pRBD had cognitive impairment in global cognition as well as in several domains involving memory, visuospatial and executive functions. The PD-pRBD subgroup showed worse performance in all neuropsychological tests with respect to controls of similar age and education. Compared with the control group, PD-pRBD showed statistically significant differences in MoCA, semantic and phonemic fluency, SDMT, JLO, HVLT-R immediate and delayed recall. In contrast, PD-non pRBD patients differed from controls only in MoCA and SDMT, although they were similar to PD-pRBD in age, education, and clinical characteristics. The results between PD-pRBD and PD-non pRBD group remain when controlling for MDS-UPDRS score. The PD subgroups comparison showed statistical differences in semantic fluency and JLO. More severe cognitive impairment in de novo PD patients with RBD symtomps has been previously reported^6,7^. This severe cognitive impairment in PD-pRBD is in accordance with the significant differences compared with healthy controls that we obtained in the three measures of global atrophy showing reduced cortical and subcortical GM, and ventricular volumes enlargement. PD-non pRBD only differed from controls in reduced cortical GM.

Cortical thickness analyses did not show significant differences between PD patients with and without pRBD. Regarding the analyses of cortical thickness maps, we found thinning in the right superior temporal gyrus extended to middle and inferior temporal gyri in PD-pRBD compared with controls. Similarly, recent studies found significant thinning in the right inferior temporal gyrus in PD-RBD compared with PD-non RBD^15^, as well as in PD-pRBD in the bilateral inferior temporal gyrus compared with PD-non pRBD^12^, both using smaller samples and the second in absence of a healthy control group to perform comparisons.

In addition to the greater global atrophy measures, we also found that PD-pRBD had a reduction of several subcortical GM structures. Comparably to previous reports using different data analysis approaches^10,11^ we found atrophy in the putamen and amygdala, but we also identified reductions in the hippocampus. The hippocampal atrophy is also coherent with the memory impairment identified in our study, even if we did not find significant correlations or regression models in this regard. iRBD is considered a prodromal symptom of alpha-synucleinopathies, thus in part comparable with PD-pRBD. In this context, it has been reported reductions in bilateral and right putamen^16,17^ and hippocampus^18^.

The contrast between both PD samples showed that PD-pRBD had lower left thalamus volume compared with PD-non pRBD. Similar findings regarding thalamic reductions associated with RBD in PD were reported with other MRI methodological approaches^10,13^. In the same line, correlation analyses also reinforce the role of the thalamus in subjects with RBD symptomatology. It has been found a negative correlation between the severity of RBD symptoms and bilateral thalamic volume^11^. PET studies also pointed to the relevance of thalamic structures linked to this symptomatology. Concretely, a recent PET study in iRBD found an increased cholinergic innervation in some nuclei of the brainstem and the ventromedial area of the thalamus^19^. This finding raises the possibility of an initial compensatory cholinergic activity in the prodromal phase of PD that would be behind the volume reduction too, even more, considering our sample is formed by newly diagnosed drug naïve PD cases. Thalamic volume decrease in our study might be explained in this way.

The differences in subcortical GM measures reflected a left-lateralized pattern in the PD-pRBD group. Previous neuroimaging research has reported structural and functional left asymmetries in PD and iRBD patients. For example, predominant left-hemispheric findings in cortical thinning in early PD^20^, in reductions of structural connectivity in PD-MCI^21^, and reductions of functional connectivity in iRBD^22^. The origin of this pattern of degeneration is unknown. However, the absence or minority of significant results in the right hemisphere does not necessarily imply that the right hemisphere is unaffected. Thus, the specific threshold established for statistical significance could contribute to explain this fact.

We observed significant correlations between neuropsychological and brain atrophy measures. To minimize the effects of multiple comparisons we performed two regression analyses, one focused on global measures of brain atrophy and the other only including the subcortical GM volumes. In the whole de novo PD group, we found that subcortical gray matter and ventricular system volumes, as well as of the left amygdala, bilateral putamen, left thalamus, and left hippocampus volumes were related with cognition. Regarding the PD-pRBD group, the worst performance in MoCA was predicted by reductions of global subcortical gray matter volume and specifically by reductions of the right putamen and left hippocampus. The impairment of speed of mental processing assessed by SDMT was related with subcortical gray matter and left hippocampus reductions. Visuospatial function impairment measured by JLO was related with the left thalamus reduction and phonetic fluency by cortical gray matter and left putamen volume. These data reinforce the value of aforesaid tests to identify early brain degeneration in de novo PD patients, in special those with more brain atrophy and cognitive impairment such as patients with RBD symptomatology. Supporting the relevance of these tests, a longitudinal study in de novo PD patients found that baseline RBD was associated with a greater annual rate of decline in MoCA and SDMT scores^7^. To our knowledge, no previous study relate MRI and neuropsychological findings in de novo PD with probable RBD. We report that cognitive impairment was mainly related with subcortical gray matter reductions.

The main strength of our study was the large sample of de novo PD patients that precludes pharmacological effects on cognitive performance as well as the effects of progressive brain atrophy involving widespread cortical atrophy. Moreover, we studied subcortical and cortical brain atrophy and cognition in a large sample of PD-pRBD, as well as studying cortical thickness differences in a large sample of de novo PD including PD-pRBD as a group. However, our study has two main limitations. First, and important, is that the diagnosis of RBD was carried out by means of a validated questionnaire but was not confirmed through vPSG. Second, PPMI is a multicenter cohort study thus there are evident differences in MRI acquisition, some of them of 1.5 Tesla. Last, even though the final sample was large, around 31% of the available MRI scans of PD patients were discarded after initial quality control, mainly due to motion artifacts and associated problems of registration, skull stripping, segmentation, and cortical surface reconstruction after preprocessing. This fact could affect the representativeness of the results.

From the whole data analyses, we can conclude that de novo PD patients with probable RBD show worse cognitive performance than those PD patients without probable RBD. The greater neuropsychological impairment is coherent with signs of global brain atrophy. Moreover, this subgroup of early PD with probable RBD shows decreased volumes in specific deep gray matter nuclei involving the amygdala, hippocampus, thalamus, and basal ganglia. The main difference between both PD subgroups is seen in the thalamus. Lastly, cognitive impairment is essentially related with subcortical gray matter reductions.

## Methods

### Participants

Data were obtained from the PPMI database (http://www.ppmi-info.org)^23^. T1-weighted images, clinical and neuropsychological data obtained from 205 newly diagnosed drug naïve PD patients, and 69 healthy controls were included. We divided PD patients into two groups, 79 PD-pRBD and 126 PD-non pRBD patients, based on available data from RBDSQ, with a 5-points cutoff^24^. All imaging and clinical data were acquired before any L-DOPA intake. All participating PPMI sites received approval from an ethical standards committee prior to study initiation, for a list of participant sites see https://www.ppmi-info.org/about-ppmi/ppmi-clinical-sites. Central IRB aprroval provided by CWG IRB (tracking number: 20200597; current clinical trial identifier of PPMI study in clinicaltrials.gov: NCT04477785). All participants provided written informed consent. Inclusion criteria were: (1) recent diagnosis of PD with asymmetric resting tremor or asymmetric bradykinesia, or two of: bradykinesia, resting tremor, and rigidity; (2) absence of treatment for PD; (3) neuroimaging evidence of significant dopamine transporter deficit consistent with the clinical diagnosis of PD and ruling out PD lookalike conditions such as drug-induced and vascular parkinsonism or essential tremor; (4) available T1-weighted images (for both PD patients and controls); and (5) age between 50 and 85 years old (for both PD patients and controls). Exclusion criteria for all participants were: (1) diagnosis of dementia; (2) significant psychiatric, neurologic, or systemic comorbidity; (3) first-degree family member with PD; and (4) presence of MRI motion artifacts, field distortions, intensity inhomogeneities, or detectable structural brain lesions. We include a detailed flow diagram of sample selection, in which we reflect the process after consulting the PPMI clinical databases and preprocessing MRI images, beyond the inclusion criteria applied by the research centers (Supplementary Fig. 1).

### Clinical and neuropsychological assessments

PD symptoms were assessed with the MDS-UPDRS, motor symptoms with the MDS-UPDRS Part III, and disease severity with H&Y. Global cognition was assessed with the MoCA, depressive symptoms using the GDS-15 (with a 5-points cutoff for depression), olfactory function using the UPSIT-40, and presence of constipation with the item 6 of the Scales for SCOPA-AUT (with a 1-point cutoff for constipation). The presence of pRBD status was assessed using the RBDSQ^24^, and the occurrence of excessive daytime sleepiness using the ESS (with a 10-points cutoff for sleepiness)^23^.

All subjects underwent a neuropsychological battery including HVLT-R, JLO short form (15-item version), SDMT, LNS, phonemic (letter ‘f’), and semantic (animals, fruits, and vegetables) verbal fluency^23^. All neuropsychological tasks were z-scored adjusted by age, sex, and education taking the control group as reference, as previously described in Segura et al.^25^.

### MRI acquisition and preprocessing

T1-weighted MRI scans were acquired using 1.5 or 3 Tesla scanners at different centers using MPRAGE sequences. Typical MRI parameters were repetition time 5–11 ms; echo time 2–6 ms; slice thickness 11.5 mm; inter-slice gap 0 mm; voxel size 1 × 1 × 1.2 mm; matrix 256 × minimum 160. There were no differences in the distribution of 1.5 and 3 Tesla MRI acquisitions between-groups (*Χ*^2^ = 1.933, *p* = 0.380; Supplementary Table 9).

Global atrophy measures including total cortical GM, total subcortical GM and estimated total intracranial volume (eTIV); ventricular system volume; as well as deep GM structures^26^. Volume ratios using eTIV were calculated to perform global and partial volumetric analyses ((volume/eTIV) * 100). Cortical thickness was estimated using FreeSurfer version 6.0 (https://surfer.nmr.mgh.harvard.edu) specific tools and the automated stream. Detailed information about the processing FreeSurfer stream is described in Uribe et al.^27^. After preprocessing, results for each subject were visually inspected to ensure accuracy of registration, skull stripping, segmentation, and cortical surface reconstruction. Possible errors were fixed by manual intervention following standard procedures (applied corrections are specified in Supplementary Fig. 1).

### Statistical analyses

Group differences in demographic, neuropsychological, clinical, and volumetric variables were conducted using IBM SPSS Statistics 25.0.0 (2017; Armonk, NY: IBM Corp) using analysis of variance (ANOVA) followed by post hoc test corrected by Bonferroni or Games-Howell. Pearson’s *Χ*^2^ tests were used for categorical measures. Correlation analyses between structural and neuropsychological variables were also conducted. Statistical significance threshold was set at *p* < 0.05.

Multiple linear regression analyses were performed using RStudio 1.1.1093 (2020; Boston, MA: RStudio PBC). As a response variable, each model included a neuropsychological variable showing significant differences in the intergroup comparisons between PD-pRBD and one of the other two groups, PD-non pRBD or controls. We tested, in the PD-pRBD group, models including global (model 1) or partial volume ratios (model 2) with a significant reduction in PD-pRBD group as predictors separately. Additionally, we tested these models in the de novo PD group, as a whole. A stepwise model selection by Akaike information criterion (AIC) was applied to the multiple linear regression models to pick the best-fitted model^28^. Only models with statistical significance threshold set at *p* < 0.05 were reported.

Intergroup cortical thickness comparisons were performed using a vertex-by-vertex general linear model with FreeSurfer version 6.0. The model included cortical thickness as a dependent factor and group as an independent factor. All results were corrected for multiple comparisons using a pre-cached cluster-wise Monte Carlo simulation with 10,000 iterations. Reported cortical regions reached a two-tailed corrected significance level of *p* < 0.05.

### Reporting summary

Further information on research design is available in the Nature Research Reporting Summary linked to this article.

## Supplementary information

Supplementary Material

Reporting Summary Checklist

## Data Availability

Data used in the preparation of this article were obtained from the Parkinson’s Progression Markers Initiative (PPMI) database (https://www.ppmi-info.org/access-data-specimens/download-data). For up-to-date information on the study, visit http://www.ppmi-info.org.
