# Supplementary material for: Brain atrophy pattern in de novo Parkinson’s disease with probable RBD associated with cognitive impairment
Source: NPJ Parkinsons Dis. 2022 May 24;8:60. doi: 10.1038/s41531-022-00326-7 (PMC9130201; doi:10.1038/s41531-022-00326-7)
Supplement: Supplementary file 1 — Supplementary Material [file 41531_2022_326_MOESM1_ESM.pdf]

SUPPLEMENTARY FIGURES

Supplementary Figure 1. Flow diagram of sample selection. Abbreviations: CP = control points; FS = FreeSurfer; HC = healthy controls; ME = manual erase; PD = Parkinson's disease; QC = quality control.

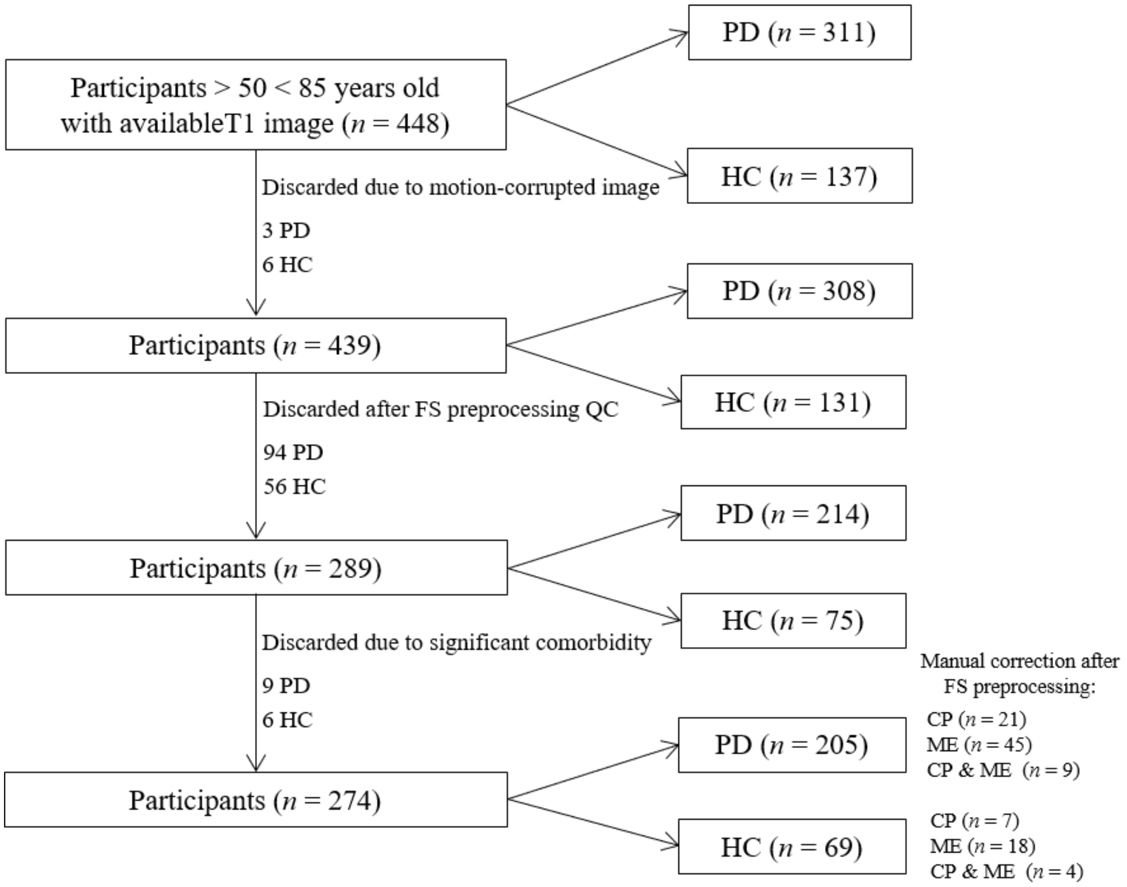

Supplementary Figure 2. Cortical thickness differences between HC and PD-pRBD. Color maps indicate significant differences (corrected $p < 0.05$). Results were corrected by Monte Carlo simulation. Abbreviations: HC = healthy controls; PD-pRBD = PD with probable RBD.

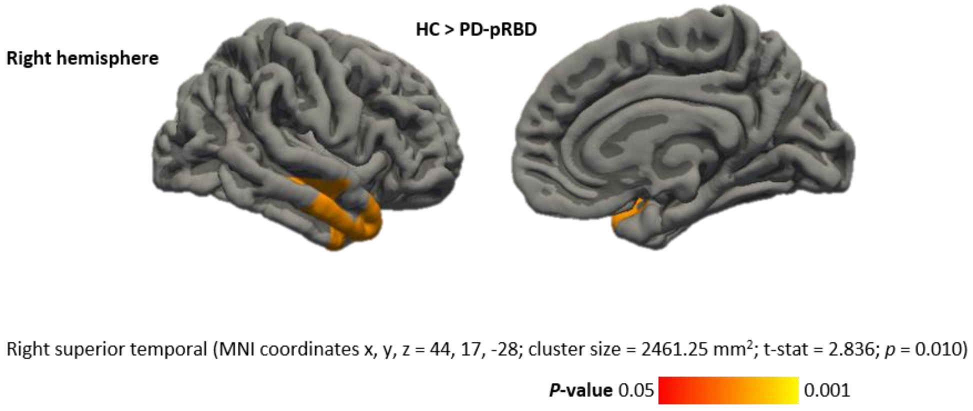

SUPPLEMENTARY TABLES

Supplementary Table 1

Neuropsychological tasks scores of PD-non pRBD, PD-pRBD and HC

	PD-non pRBD	PD-pRBD	HC	Test stats	*P*-value
MoCA	27.0 (2.2)	26.8 (2.1)	28.1 (1.1)	9.105[1]	<0.001[3, 4]
Semantic fluency					
Animals	21.8 (5.2)	19.5 (4.4)	22.7 (5.4)	5.623[2]	0.004[4, 5]
Vegetables	14.7 (4.4)	13.6 (4.0)	15.4 (3.7)	1.498[2]	0.226
Fruits	14.3 (4.1)	13.1 (4.0)	15.5 (4.2)	2.924[2]	0.055[4]
Phonetic fluency ‘f’	12.9 (4.7)	12.1 (4.2)	14.3 (4.5)	4.439[2]	0.013[4]
SDMT	42.0 (8.3)	38.9 (9.7)	46.6 (8.9)	12.939[2]	<0.001[3, 4]
LNS	10.8 (2.6)	9.9 (2.4)	11.3 (2.6)	5.422[2]	0.005[4]
JLO	13.1 (1.9)	12.5 (2.2)	13.3 (1.7)	4.019[2]	0.019[4, 5]
HVLT-R Immediate recall	25.0 (5.0)	23.3 (4.7)	25.9 (4.4)	3.465[2]	0.033[4]
HVLT-R Recognition	9.6 (2.9)	9.2 (3.0)	9.9 (3.2)	1.708[2]	0.183
HVLT-R Delayed recall	8.5 (2.4)	7.7 (2.6)	9.2 (2.4)	4.528[2]	0.012[4]

Abbreviations: MoCA = Montreal Cognitive Assessment; SDMT = Symbol Digit Modalities Test; LNS = Letter-Number Sequencing; JLO = Benton Judgment of Line Orientation; HVLT-R = Hopkins Verbal Learning Test-Revised; PD-non pRBD = PD without probable RBD; PD-pRBD = PD with probable RBD; HC = healthy controls.

Data are presented by groups as mean (SD). Analyses were conducted on z-scores adjusted by age, sex and education.

[1] Analysis of variance (ANOVA) followed post hoc test corrected by Games-Howell was used.

[2] Analysis of variance (ANOVA) followed by post hoc test corrected by Bonferroni was used.

[3] Significant differences ($p < 0.05$) were found between PD-non pRBD and HC.

[4] Significant differences ($p < 0.05$) were found between PD-pRBD and HC.

[5] Significant differences ($p < 0.05$) were found between PD-non pRBD and PD-pRBD.

Supplementary Table 2

Neuropsychological tasks scores of PD-non pRBD and PD-pRBD analyzed with MDS-UPDRS score as a covariate

	**PD-non pRBD**	**PD-pRBD**	**Test stats**	***P*-value**
MoCA	27.0 (2.2)	26.8 (2.1)	0.135	0.713
Semantic fluency				
Animals	21.8 (5.2)	19.5 (4.4)	7.737	0.006[1]
Vegetables	14.7 (4.4)	13.6 (4.0)	1.215	0.272
Fruits	14.3 (4.1)	13.1 (4.0)	1.415	0.236
Phonetic fluency ‘f’	12.9 (4.7)	12.1 (4.2)	2.872	0.092
SDMT	42.0 (8.3)	38.9 (9.7)	1.756	0.187
LNS	10.8 (2.6)	9.9 (2.4)	4.553	0.034[1]
JLO	13.1 (1.9)	12.5 (2.2)	4.434	0.036[1]
HVLT-R Immediate recall	25.0 (5.0)	23.3 (4.7)	2.423	0.121
HVLT-R Recognition	9.6 (2.9)	9.2 (3.0)	0.515	0.474
HVLT-R Delayed recall	8.5 (2.4)	7.7 (2.6)	3.529	0.062

Abbreviations: MoCA = Montreal Cognitive Assessment; SDMT = Symbol Digit Modalities Test; LNS = Letter-Number Sequencing; JLO = Benton Judgment of Line Orientation; HVLT-R = Hopkins Verbal Learning Test-Revised; PD-non pRBD = PD without probable RBD; PD-pRBD = PD with probable RBD.

Data are presented by groups as mean (SD). Analyses were conducted on z-scores adjusted by age, sex and education.

Analysis of covariance (ANCOVA) with MDS-UPDRS as a covariate followed post hoc test corrected by Bonferroni was used.

[1] Significant differences ($p < 0.05$) were found between PD-non pRBD and PD-pRBD.

Supplementary Table 3

Global and partial volume ratios of PD-non pRBD and PD-pRBD analyzed with MDS-UPDRS score as a covariate

	**PD-non pRBD**	**PD-pRBD**	**Test stats**	***P*-value**
**Global volumes**				
Cortical GM	28.6704 (2.16624)	28.7482 (2.51696)	0.280	0.598
Subcortical GM	3.5597 (0.25621)	3.4926 (0.29242)	2.187	0.141
Ventricular system	1.9743 (0.99272)	2.2429 (1.04248)	2.604	0.108
**Deep GM nuclei volumes**				
Left Thalamus	0.4559 (0.04486)	0.4407 (0.04097)	4.780	0.030[1]
Right Thalamus	0.4460 (0.04276)	0.4348 (0.04267)	2.183	0.141
Left Caudate	0.2124 (0.02607)	0.2108 (0.02504)	0.035	0.851
Right Caudate	0.2190 (0.02797)	0.2167 (0.02808)	0.116	0.734
Left Putamen	0.2902 (0.03366)	0.2811 (0.03785)	2.081	0.151
Right Putamen	0.2883 (0.03211)	0.2816 (0.03791)	1.159	0.283
Left Pallidum	0.1280 (0.01427)	0.1259 (0.01687)	1.105	0.294
Right Pallidum	0.1256 (0.01355)	0.1212 (0.01663)	6.095	0.014[1]
Left Hippocampus	0.2548 (0.02980)	0.2494 (0.02969)	1.487	0.224
Right Hippocampus	0.2619 (0.03127)	0.2606 (0.03106)	0.027	0.870
Left Amygdala	0.1000 (0.01482)	0.0984 (0.01815)	0.327	0.568
Right Amygdala	0.1089 (0.01427)	0.1088 (0.01671)	0.000	0.991
Left Accumbens	0.0294 (0.00680)	0.0300 (0.00732)	0.969	0.326
Right Accumbens	0.0317 (0.00662)	0.0317 (0.00700)	0.037	0.848
Brainstem	1.3904 (0.12004)	1.3618 (0.12659)	3.096	0.080

Abbreviations: GM = gray matter; PD-non pRBD = PD without probable RBD; PD-pRBD = PD with probable RBD.

Data are presented by groups as mean (SD). Analysis of covariance (ANCOVA) with MDS-UPDRS score as covariate followed by post hoc test corrected by Bonferroni was used.
[1] Significant differences ($p < 0.05$) were found between PD-non pRBD and PD-pRBD.

Supplementary Table 4

Global and partial volumes of PD-non pRBD, PD-pRBD and HC

	PD-non pRBD	PD-pRBD	HC
**eTIV**	1597513.1 (168028.4)	1585321.3 (182638.3)	1517584.7 (161845.2)
**Global volumes**			
Cortical GM	456759.5 (49193.7)	453153.9 (44220.5)	449381.8 (46580.6)
Subcortical GM	56683.9 (5676.9)	55031.7 (4772.8)	54383.8 (4176.4)
Ventricular system	32091.7 (18125.7)	36245.6 (21389.0)	27705.2 (14698.7)
**Deep GM nuclei volumes**			
Left Thalamus	7267.1 (944.1)	6964.1 (869.3)	6880.9 (696.7)
Right Thalamus	7107.0 (891.9)	6873.6 (891.5)	6729.9 (771.9)
Left Caudate	3381.5 (483.9)	3321.9 (413.4)	3255.8 (411.9)
Right Caudate	3488.5 (527.4)	3418.6 (510.0)	3326.6 (433.9)
Left Putamen	4626.5 (644.9)	4423.5 (558.2)	4453.8 (502.9)
Right Putamen	4588.9 (585.8)	4423.6 (514.2)	4450.6 (447.5)
Left Pallidum	2039.9 (271.4)	1978.6 (227.1)	1912.8 (227.7)
Right Pallidum	2002.1 (271.9)	1904.7 (217.1)	1866.1 (212.2)
Left Hippocampus	4050.0 (498.6)	3919.0 (377.0)	3950.5 (355.4)
Right Hippocampus	4159.7 (504.9)	4095.1 (397.8)	4067.7 (377.8)
Left Amygdala	1593.3 (266.9)	1543.5 (228.1)	1585.4 (198.0)
Right Amygdala	1733.7 (263.5)	1709.1 (220.2)	1728.0 (187.7)
Left Accumbens	468.1 (110.9)	471.2 (108.4)	452.1 (91.0)
Right Accumbens	504.4 (106.2)	498.3 (103.5)	486.2 (84.6)
Brainstem	22154.5 (2587.9)	21523.3 (2708.2)	20963.2 (2149.7)

Abbreviations: eTIV = estimated total intracranial volume; GM = gray matter; PD-non pRBD = PD without probable RBD; PD-pRBD = PD with probable RBD; HC = healthy controls.

Data are presented by groups as mean (SD) in mm^3.

Supplementary Table 5

Bivariate significative correlations between MRI measures and performance in neuropsychological tasks in the PD-pRBD group

	Global MRI measures	**r (*P*-value)**	***Partial MRI measures***	**r (*P*-value)**
MoCA	Cortical GM	0.229 (0.042)	Left Putamen	0.334 (0.003)
	Subcortical GM	0.350 (0.002)	Right Putamen	0.368 (<0.001)
			Left Hippocampus	0.362 (0.001)
			Left Amygdala	0.284 (0.011)
Phonetic fluency ‘f’	Cortical GM	0.348 (0.002)	Left Putamen	0.272 (0.015)
	Subcortical GM	0.269 (0.017)	Right Putamen	0.266 (0.018)
SDMT	Cortical GM	0.247 (0.030)	Right Putamen	0.236 (0.038)
	Subcortical GM	0.272 (0.016)	Left Hippocampus	0.302 (0.007)
	Ventricular system	-0.267 (0.018)	Left Amygdala	0.241 (0.034)
JLO			Left Thalamus	0.296 (0.009)

Abbreviations: GM = gray matter; MoCA = Montreal Cognitive Assessment; SDMT = Symbol Digit Modalities Test; JLO = Benton Judgment of Line Orientation.

Supplementary Table 6

Bivariate significative correlations between MRI measures and performance in neuropsychological tasks in the whole PD group

	Global MRI measures	**r (*P*-value)**	***Partial MRI measures***	**r (*P*-value)**
MoCA	Cortical GM	0.209 (0.003)	Left Putamen	0.225 (0.001)
	Subcortical GM	0.221 (0.001)	Right Putamen	0.240 (<0.001)
			Left Hippocampus	0.178 (0.011)
			Left Amygdala	0.215 (0.002)
Semantic fluency Animals	Subcortical GM	0.163 (0.020)	Left Amygdala	0.184 (0.009)
			Right superior temporal	0.171 (0.015)
Semantic fluency Fruits	Ventricular system	-0.167 (0.017)	Left Amygdala	0.142 (0.044)
			Right superior temporal	0.169 (0.016)
Phonetic fluency ‘f’	Cortical GM	0.141 (0.043)	Left Thalamus	0.176 (0.012)
	Subcortical GM	0.175 (0.012)	Right Putamen	0.179 (0.010)
			Left Hippocampus	0.139 (0.048)
			Left Amygdala	0.198 (0.004)
SDMT	Cortical GM	0.166 (0.018)	Left Thalamus	0.192 (0.006)
	Subcortical GM	0.246 (<0.001)	Left Putamen	0.166 (0.018)
	Ventricular system	-0.237 (<0.001)	Right Putamen	0.186 (0.008)
			Left Hippocampus	0.192 (0.004)
			Left Amygdala	0.181 (0.010)
LNS			Left Thalamus	0.189 (0.007)
JLO	Ventricular system	-0.156 (0.026)		
HVLT-R Immediate recall	Subcortical GM	0.141 (0.045)	Left Hippocampus	0.171 (0.015)
	Ventricular system	-0.179 (0.011)	Left Amygdala	0.156 (0.026)
			Right superior temporal	0.196 (0.005)
HVLT-R Delayed recall	Subcortical GM	0.186 (0.008)	Left Thalamus	0.219 (0.002)
	Ventricular system	-0.211 (0.003)	Left Hippocampus	0.160 (0.022)
			Left Amygdala	0.148 (0.035)
			Right superior temporal	0.150 (0.032)

Abbreviations: GM = gray matter; MoCA = Montreal Cognitive Assessment; SDMT = Symbol Digit Modalities Test; LNS = Letter-Number Sequencing; JLO = Benton Judgment of Line Orientation; HVLT-R = Hopkins Verbal Learning Test-Revised.

Supplementary Table 7

Multiple regression results of partial and global volume ratios as predictors of performance in neuropsychological tasks in the whole PD group

	Model 1 Global MRI measures		*Model 2 Partial MRI measures*	
	**variables**	**t-stat (P-value)**	**variables**	**t-stat (P-value)**
MoCA	Subcortical GM	3.113 (0.002)	Right Putamen	2.539 (0.012)
			Left Amygdala	2.107 (0.036)
Semantic fluency Animals	Subcortical GM	2.338 (0.020)	Left Amygdala	2.647 (0.009)
Semantic fluency Fruits	Ventricular system	-2.484 (0.014)	Right Putamen	-2.250 (0.025)
			Left Amygdala	2.453 (0.015)
Phonetic fluency ‘f’	Subcortical GM	3.335 (0.001)	Left Thalamus	2.383 (0.018)
			Left Amygdala	2.304 (0.022)
SDMT	Subcortical GM	2.277 (0.024)	Left Thalamus	2.190 (0.030)
			Left Amygdala	1.981 (0.049)
LNS	Non-significant model		Left Thalamus	3.202 (0.002)
JLO	Non-significant model		Non-significant model	
HVLT-R Immediate recall	Ventricular system	-2.585 (0.010)	Left Hippocampus	2.922 (0.004)
HVLT-R Delayed recall	Ventricular system	-3.445 (<0.001)	Left Thalamus	1.980 (0.049)

Abbreviations: GM = gray matter; MoCA = Montreal Cognitive Assessment; SDMT = Symbol Digit Modalities Test; LNS = Letter-Number Sequencing; JLO = Benton Judgment of Line Orientation; HVLT-R = Hopkins Verbal Learning Test-Revised.

Supplementary Table 8

Adjustment of multiple regression results of partial and global volume ratios as predictors of performance in neuropsychological tasks in the whole PD group

	Model 1 Global MRI measures				*Model 2 Partial MRI measures*			
	R2	**Adjusted *R2***	***F***	***P*-value**	***R2***	**Adjusted *R2***	***F***	***P*-value**
MoCA	0.046	0.041	9.691	0.002	0.089	0.080	9.869	<0.001
Semantic fluency Animals	0.026	0.022	5.465	0.020	0.034	0.029	7.007	0.009
Semantic fluency Fruits	0.030	0.025	6.172	0.014	0.050	0.036	3.495	0.017
Phonetic fluency ‘f’	0.052	0.047	11.120	0.001	0.069	0.060	7.465	<0.001
SDMT	0.074	0.064	7.958	<0.001	0.055	0.046	5.870	0.003
LNS					0.049	0.039	5.131	0.007
JLO								
HVLT-R Immediate recall	0.032	0.027	6.680	0.010	0.041	0.036	8.539	0.004
HVLT-R Delayed recall	0.056	0.051	11.870	<0.001	0.055	0.046	5.860	0.003

Abbreviations: GM = gray matter; MoCA = Montreal Cognitive Assessment; SDMT = Symbol Digit Modalities Test; JLO = Benton Judgment of Line Orientation; LNS = Letter-Number Sequencing; HVLT-R = Hopkins Verbal Learning Test-Revised.

Supplementary Table 9

MRI field strength distribution of the groups

	1.5 T	3 T	Test stat (*P*-value)
PD-non pRBD	37 (29.4%)	89 (70.6%)	1.933 (0.380)
PD-pRBD	20 (25.3%)	59 (74.7%)	
HC	14 (20.3%)	55 (79.7%)	

Abbreviations: T = Tesla; PD-non pRBD = PD without probable RBD; PD-pRBD = PD with probable RBD; HC = healthy controls.

Data are presented by groups as *n* (%). Pearson's chi-squared was used.
