# Supplementary material for: Brain atrophy pattern in de novo Parkinson’s disease with probable RBD associated with cognitive impairment
Source: NPJ Parkinsons Dis. 2022 May 24;8:60. doi: 10.1038/s41531-022-00326-7 (PMC9130201; doi:10.1038/s41531-022-00326-7)
Supplement: Supplementary file 2 — Reporting Summary Checklist [file 41531_2022_326_MOESM2_ESM.pdf]

nature portfolio

Corresponding author(s): DBPR NPJPARKD-01008R2

Last updated by author(s): Apr 8, 2022

Reporting Summary

Nature Portfolio wishes to improve the reproducibility of the work that we publish. This form provides structure for consistency and transparency in reporting. For further information on Nature Portfolio policies, see our Editorial Policies and the Editorial Policy Checklist.

Statistics

For all statistical analyses, confirm that the following items are present in the figure legend, table legend, main text, or Methods section.

n/a	Confirmed	
☐	☒	The exact sample size (n) for each experimental group/condition, given as a discrete number and unit of measurement
☐	☒	A statement on whether measurements were taken from distinct samples or whether the same sample was measured repeatedly
☐	☒	The statistical test(s) used AND whether they are one- or two-sided<br>*Only common tests should be described solely by name; describe more complex techniques in the Methods section.*
☐	☒	A description of all covariates tested
☐	☒	A description of any assumptions or corrections, such as tests of normality and adjustment for multiple comparisons
☐	☒	A full description of the statistical parameters including central tendency (e.g. means) or other basic estimates (e.g. regression coefficient) AND variation (e.g. standard deviation) or associated estimates of uncertainty (e.g. confidence intervals)
☒	☐	For null hypothesis testing, the test statistic (e.g. F, t, r) with confidence intervals, effect sizes, degrees of freedom and P value noted<br>*Give P values as exact values whenever suitable.*
☒	☐	For Bayesian analysis, information on the choice of priors and Markov chain Monte Carlo settings
☒	☐	For hierarchical and complex designs, identification of the appropriate level for tests and full reporting of outcomes
☒	☐	Estimates of effect sizes (e.g. Cohen's d, Pearson's r), indicating how they were calculated

Our web collection on statistics for biologists contains articles on many of the points above.

Software and code

Policy information about availability of computer code

Data collection	n/a
Data analysis	FreeSurfer version 6.0., IBM SPSS Statistics 25.00.0, RStudio 1.1.1093

For manuscripts utilizing custom algorithms or software that are central to the research but not yet described in published literature, software must be made available to editors and reviewers. We strongly encourage code deposition in a community repository (e.g. GitHub). See the Nature Portfolio guidelines for submitting code & software for further information.

Data

Policy information about availability of data

All manuscripts must include a data availability statement. This statement should provide the following information, where applicable:

- Accession codes, unique identifiers, or web links for publicly available datasets
- A description of any restrictions on data availability
- For clinical datasets or third party data, please ensure that the statement adheres to our policy

Data used in the preparation of this article wew obtained from the Parkinson's Progression Markers Initiative (PPMI) database. For up-to-date information on the study, visit https://www.ppmi-info.org/ .

Field-specific reporting

Please select the one below that is the best fit for your research. If you are not sure, read the appropriate sections before making your selection.

☒ Life sciences ☐ Behavioural & social sciences ☐ Ecological, evolutionary & environmental sciences

For a reference copy of the document with all sections, see nature.com/documents/nr-reporting-summary-flat.pdf

Life sciences study design

All studies must disclose on these points even when the disclosure is negative.

Sample size	T1-weighted images, clinical and neuropsychological data obtained from 205 newly diagnosed drug naïve PD patients, and 69 healthy controls were included. We divided PD patients into two groups, 79 PD-pRBD and 126 PD-non pRBD patients, based on available data from RBDSQ.
Data exclusions	Exclusion: (1) diagnosis of dementia; (2) significant psychiatric, neurologic or systemic comorbidity; (3) first-degree family member with PD; and (4) presence of MRI motion artifacts, field distortions, intensity inhomogeneities, or detectable structural brain lesions.
Replication	Data from PPMI is available online .
Randomization	We used MDS-Unified Parkinson's Disease Rating Scale (MDS-UPDRS) score as a covariate.
Blinding	Blinding no necesary because no intervation was carried out.

Reporting for specific materials, systems and methods

We require information from authors about some types of materials, experimental systems and methods used in many studies. Here, indicate whether each material, system or method listed is relevant to your study. If you are not sure if a list item applies to your research, read the appropriate section before selecting a response.

Materials & experimental systems

n/a	Involved in the study	
☒	☐	Antibodies
☒	☐	Eukaryotic cell lines
☒	☐	Palaeontology and archaeology
☒	☐	Animals and other organisms
☐	☒	Human research participants
☐	☒	Clinical data
☒	☐	Dual use research of concern

Methods

n/a	Involved in the study	
☒	☐	ChIP-seq
☒	☐	Flow cytometry
☐	☒	MRI-based neuroimaging

Human research participants

Policy information about studies involving human research participants

Population characteristics	See above.
Recruitment	Multicenter study in which all participating PPMI sites received approval from an ethical standards committee prior to study initiation.
Ethics oversight	Ethical standards committee approval prior to study initiation and WCG IRB Connexus approval to PPMI.

Note that full information on the approval of the study protocol must also be provided in the manuscript.

Clinical data

Policy information about clinical studies

All manuscripts should comply with the ICMJE guidelines for publication of clinical research and a completed CONSORT checklist must be included with all submissions.

Clinical trial registration	NCT04477785
Study protocol	Specified in: https://www.ppmi-info.org/study-design/research-documents-and-sops .
Data collection	Specified in: https://www.ppmi-info.org/study-design/research-documents-and-sops .

Outcomes | Specified in: https://www.ppmi-info.org/study-design/research-documents-and-sops .

Magnetic resonance imaging

Experimental design

Design type: Structural MRI T1-based study.

Design specifications: n/a

Behavioral performance measures: n/a

Acquisition

Imaging type(s): Structural.

Field strength: 1.5 and 3T.

Sequence & imaging parameters: T1-weighted MRI scans were acquired using 1.5 or 3 Tesla scanners at different centers using MPRAGE sequences. Typical MRI parameters were repetition time 5-11 ms; echo time 2-6 ms; slice thickness 1-1.5 mm; inter-slice gap 0 mm; voxel size 1x1x1.2 mm; matrix 256 x minimum 160.

Area of acquisition: Whole brain.

Diffusion MRI ☐ Used ☒ Not used

Preprocessing

Preprocessing software:

FreeSurfer v.6.0.:

"Cortical thickness was estimated using the automated FreeSurfer stream (version 6.0). The procedures carried out by FreeSurfer include removal of non-brain data, intensity normalization (Fischl et al., 2001), tessellation of the GM / white matter (WM) boundary, automated topology correction (Dale et al., 1999; Ségonne et al., 2007), and accurate surface deformation to identify tissue borders (Dale and Sereno, 1993; Fischl and Dale, 2000; Fischl et al., 2002). Cortical thickness is then calculated as the distance between the WM and GM surfaces at each vertex of the reconstructed cortical mantle (Fischl et al., 2002). After FreeSurfer preprocessing, results for each subject were visually inspected to ensure accuracy of registration, skull stripping, segmentation, and cortical surface reconstruction. Maps were smoothed using a circularly symmetric Gaussian kernel across the surface with a full width at half maximum (FWHM) of 15 mm." (Uribe et al., 2019)

Global atrophy measures including total cortical gray matter (GM), total subcortical GM and estimated total intracranial volume (eTIV); ventricular system volume; as well as deep GM structures using AsegAtlas: https://surfer.nmr.mgh.harvard.edu/ftp/articles/fischl02-labeling.pdf. Volume ratios using eTIV were calculated to perform global and partial volumetric analyses ((volume / eTIV) * 100).

References:

Fischl B., Liu A., & Dale A. M., Automated manifold surgery: constructing geometrically accurate and topologically correct models of the human cerebral cortex, IEEE Trans Med Imaging, 20, 70–80, (2001).

Dale, A. M., Fischl, B., & Sereno, M. I., Cortical surface-based analysis: I. Segmentation and surface reconstruction, Neuroimage, 9, 179–194, (1999).

Ségonne, F., Pacheco, J., & Fischl, B., Geometrically accurate topology-correction of cortical surfaces using nonseparating loops, IEEE Trans.Med Imaging, 26, 518–529, (2007).

Dale, A. M., & Sereno, M. I., Improved localizadon of cortical activity by combining EEG and MEG with MRI cortical surface reconstruction: a linear approach, J Cogn Neurosci, 5, 162–176, (1993).

Fischl B. & Dale A. M., Measuring the thickness of the human cerebral cortex from magnetic resonance images, Proc. Natl. Acad. Sci. U.S.A., 97, 11050–11055, (2000).

Fischl B., et al., Whole brain segmentation: automated labeling of neuroanatomical structures in the human brain, Neuron 33, 341–355, (2002).

Uribe C. et al., Neuroanatomical and functional correlates of cognitive and affective empathy in young healthy adults, Front. Behav. Neurosci., https://doi.org/10.3389/fnbeh.2019.00085, (2019).

Normalization: Specifications above (Preprocessing software section).

Normalization template: Specifications above (Preprocessing software section).

Noise and artifact removal	Specifications above (Preprocessing software section).
Volume censoring	Specifications above (Preprocessing software section).

Statistical modeling & inference

Model type and settings	Group differences in demographic, neuropsychological, clinical, and volumetric variables were conducted using IBM SPSS Statistics 25.0.0 (2017; Armonk, NY: IBM Corp) using analysis of variance (ANOVA) followed by post hoc test corrected by Bonferroni or Games-Howell. Pearson's chi-squared tests were used for categorical measures. Correlation analyses between structural and neuropsychological variables were also conducted. Statistical significance threshold was set at $p < 0.05$.
Effect(s) tested	*Define precise effect in terms of the task or stimulus conditions instead of psychological concepts and indicate whether ANOVA or factorial designs were used.*

Specify type of analysis: ☐ Whole brain ☐ ROI-based ☒ Both

Anatomical location(s)	AsegAtlas: https://surfer.nmr.mgh.harvard.edu/ftp/articles/fischl02-labeling.pdf
Statistic type for inference (See Eklund et al. 2016)	n/a
Correction	Monte Carlo for cortical thickness and Bonferroni for volumetry and global MRI measures.

Models & analysis

n/a	Involved in the study
☒	☐ Functional and/or effective connectivity
☒	☐ Graph analysis
☐	☒ Multivariate modeling or predictive analysis

Multivariate modeling and predictive analysis	Multiple linear regression analyses were performed using RStudio 1.1.1093 (2020; Boston, MA: RStudio PBC). As a response variable, each model included a neuropsychological variable showing significant differences in the intergroup comparisons between PD-pRBD and one of the other two groups, PD-non pRBD or controls. We tested, in the PD-pRBD group, models including global (model 1) or partial volume ratios (model 2) with a significant reduction in PD-pRBD group as predictors separately. Additionally, we tested these models in the de novo PD group, as a whole. A stepwise model selection by Akaike information criterion (AIC) was applied to the multiple linear regression models to pick the best-fitted model. Only models with statistical significance threshold set at $p < 0.05$ were reported.
